# Changes in Early Childhood Irritability and Its Association With Depressive Symptoms and Self-Harm During Adolescence in a Nationally Representative United Kingdom Birth Cohort

**DOI:** 10.1016/j.jaac.2023.05.027

**Published:** 2024-01

**Authors:** Ramya Srinivasan, Eirini Flouri, Gemma Lewis, Francesca Solmi, Argyris Stringaris, Glyn Lewis

**Affiliations:** a UCL Division of Psychiatry, London, United Kingdom; b Emotion & Development Branch, National Institute of Mental Health, National Institutes of Health, Bethesda, Maryland; c UCL Institute of Education, London United Kingdom

**Keywords:** irritability, depression, self-harm, cohort study, MCS

## Abstract

**Objective:**

This study aimed to investigate longitudinal associations between changes in early childhood irritability, and depressive symptoms and self-harm at 14 years.

**Method:**

We used data from 7,225 children in a UK-based general population birth cohort. Childhood irritability was measured at 3, 5, and 7 years using 4 items from 2 questionnaires (the Children’s Social Behaviour Questionnaire [CSBQ] and the Strengths and Difficulties Questionnaire [SDQ]). Participants reported depressive symptoms via the short Mood and Feelings Questionnaire (sMFQ) and self-harm via a single-item question, at 14 years. We used multilevel models to calculate within-child change in irritability between 3 and 7 years and examined associations between irritability, and depressive symptoms and self-harm at 14 years using linear and logistic regression models, respectively. We adjusted for child and family sociodemographic/economic characteristics, mental health difficulties, and child cognitive development.

**Results:**

Irritability at ages 5 and 7 years was positively associated with depressive symptoms and self-harm at age 14 years. Irritability that remained high between 3 and 7 years was associated with depressive symptoms and self-harm at 14 years in unadjusted (depressive symptoms: β coefficient = 0.22, 95 % CI = 0.08-0.37, *p* = .003; self-harm: odds ratio = 1.09, 95 % CI = 1.01-1.16, *p* = .019) and adjusted models (depressive symptoms: β coefficient = 0.31, 95 % CI = 0.17-0.45, *p* < .001; self-harm: odds ratio = 1.12, 95 % CI = 1.0.4-1.19, *p* = .004). Results were similar in imputed samples.

**Conclusion:**

Children with irritability that remains high between 3 and 7 years are more likely to report higher depressive symptoms and self-harm during adolescence. These findings support early intervention for children with high irritability and universal interventions in managing irritability for parents of preschool-aged children.

Depression is a common mental health problem and leading cause of disability worldwide.^1^ Most adult depressive disorders begin in adolescence, and prevalence increases substantially during this time.^2^^,^^3^ Depression during adolescence is associated with long-term impairments.^4^ Non-suicidal self-harm (NSSH) and self-harm with intent to die (suicide attempts) also increase during adolescence and are frequently comorbid with depression.^5^^,^^6^ Despite this, little is known about the developmental mechanisms that underlie depression and self-harm; understanding these developmental processes is important in developing early preventive interventions. Early childhood is a crucial period of development with a substantial impact on mental health outcomes,^7^, ^8^, ^9^ and is therefore important to consider with regard to prevention.

Irritability is common in children^10^, ^11^, ^12^ and is defined as an “elevated proneness to anger relative to peers in response to frustration or reward omission.”^13^ Irritability, like other psychiatric symptoms, exists on a continuum. The presence of irritability during childhood can be normative and adaptive but may be considered pathological if it results in distress or impairment. Irritability is common during the preschool-age years (ages 3-5 years) and declines during childhood. There is evidence that irritability levels are highest around age 3 years, and decline steadily up to the ages of 8 or 9.^11^^,^^13^, ^14^ A slight peak in adolescence has been found followed by further decline.^15^, ^14^

Irritability is a transdiagnostic symptom, present across childhood emotional and behavioural disorders, and is a common reason for referral to child and adolescent mental health services.^16^ Although depression and self-harm might involve different risk factors, many are likely to be shared. Irritability might increase the risk of depression and self-harm through several developmental pathways. The ability to tolerate and manage frustration is a skill that allows an individual to adapt and cope with stressful situations. This might promote resilience to mental health problems, including depression and self-harm.^17^ There is evidence that parenting factors may play a role in the persistence of irritability beyond early childhood and may increase the risk of subsequent depression and self-harm.^18^ During late childhood and adolescence, as social and educational demands increase, irritability may result in children avoiding situations perceived as challenging. This could result in increased risk of depression and self-harm as children experience fewer positive experiences and social isolation. Furthermore, irritability is associated with heightened threat perception^19^; irritable children may also elicit negative social interactions,^13^ which might lead to difficulties with social isolation and adverse experiences such as bullying, which are risk factors for depression.^4^ Depression is associated with self-harm^5^^,^^6^; however, the association between irritability and self-harm may also be direct.^20^ Difficulties managing frustration may promote less adaptive coping strategies such as self-harm.

Several longitudinal studies have found that irritability is associated with an increased risk of subsequent depression in childhood and adolescence.^10^^,^^12^^,^^21^, ^22^, ^23^, ^24^, ^25^ To our knowledge, no study has investigated associations between irritability and NSSH. Compared with depression, there are also fewer studies of suicide attempts and suicidal ideation. However, there is evidence that irritability is associated with these mental health problems during adolescence.^20^^,^^26^, ^27^, ^28^

Most previous studies have focused on irritability during late childhood and adolescence. One study that examined irritability at 3 years of age found no evidence of an association with adolescent-reported depressive disorders.^25^ Changes in irritability during childhood could be more strongly associated with mental health outcomes than irritability levels at 1 point in time. This is important to consider, given the normative developmental reductions in irritability that occur during early childhood. These normative developmental changes are likely to relate to developmental advances in frustration tolerance and regulation.^10^^,^^14^, ^15^ Our hypothesis is that children whose irritability does not decrease as expected during early childhood will be at increased risk for depression and self-harm during adolescence (compared to children whose irritability is normatively high and then decreases). As far as we are aware, no studies have examined the association between changes in irritability during early childhood and subsequent depression and self-harm during adolescence. Identifying modifiable risk factors for depression and self-harm during early adolescence, when the incidence of these mental health problems peaks sharply, could inform strategies for primary prevention.

Given that irritability can be identified from preschool age, examining the relationship between changes in irritability in young children and subsequent depression and self-harm may indicate periods when early prevention might plausibly be effective, by identifying early developmental pathways that relate to future risk of depression and self-harm. This study aims to examine the relationship between changes in early childhood irritability between the ages of 3 and 7 years and adolescent depressive symptoms and self-harm. We tested the hypothesis that irritability that remains high during early childhood and does not decline in line with normative patterns would be positively associated with adolescent depression and self-harm.

## Method

### Study Design and Participants

The Millennium Cohort Study (MCS) is an ongoing population-based birth cohort of children born between 2000 and 2002, living in the United Kingdom, aged 9 months^29^ (Supplement 1, available online). Families were selected for participation from the Department of Social Security’s Child Benefit register, using a clustered stratified sampling design; MCS over-sampled those from deprived and/or ethnic minority backgrounds. As a result, the sample is designed to represent the diversity of the UK population (see https://www.closer.ac.uk/wp-content/uploads/The-Millennium-Cohort-Study-MCS-and-research-on-ethnicity.pdf for further discussion of research on ethnicity in MCS and Supplement 1, available online).

Ethics approval was given by the Multi-Centre Research Ethics Committee (MREC). We included children with complete data on all variables of interest in main analyses. For twins and triplets, 1 child was selected at random to avoid over- or under-estimation because of shared environmental or genetic factors.

### Primary Outcome: Adolescent Depressive Symptoms

The short Mood and Feelings Questionnaire (sMFQ) was completed at 14 years of age. The sMFQ is a 13-item self-report questionnaire that measures *DSM-IV* depressive symptom severity in the preceding 2 weeks.^30^ Response options are as follows: “not true” = 0, “sometimes true” = 1, “true” = 2, or missing = “don’t know” or “don’t want to answer.” Total possible scores range from 0 to 26, with higher scores reflecting higher severity. The sMFQ has been extensively validated in population-based samples of children and adolescents aged 6 to 17 years.^31^^,^^32^ It corresponds with clinical diagnostic criteria (eg, area under the curve = 0.90) and has high reliability (eg. Cronbach α = 0.9).^33^ We used continuous sMFQ scores in our primary analyses because depression is best conceptualized as a continuum in the general population.

### Secondary Outcome: Adolescent Self-Harm

Self-harm at 14 years of age was assessed based on the response to the question: “In the past year have you hurt yourself on purpose in any way?” Response options were “yes” or “no.” Those who answered “yes” were classified as having self-harmed. Brief single-item questions assessing self-harm have been widely used in large population-based studies of young people.^6^^,^^34^^,^^35^ There is evidence that, in population-based samples, young people are more likely to accurately respond to these items because of their less invasive nature.^36^^,^^37^ The self-harm question available in MCS does not differentiate between NSSH and suicide attempts. We were also not able to examine associations with suicidal ideation (thoughts or plans) because of a lack of data on this.

### Exposure: Childhood Irritability

Childhood irritability was measured using 3 questions (3 from the maternally completed Child Social Behaviour Questionnaire [CSBQ], as follows: “Is easily frustrated,” “Gets over being upset quickly,” and “Shows wide mood swings”^38^ and 1 question from the Strengths and Difficulties Questionnaire (SDQ): “Often has temper tantrums.”^39^ These measures were administered at ages 3 to 17 years. We selected ages 3, 5, and 7 years because irritability during early childhood was our main interest and these assessments preceded measurement of the outcomes (Supplement 1, available online). The 4 items were summed to create irritability scores ranging from 0 to 8, used as a continuous variable. The items were selected by a child and adolescent psychiatrist, from well-established scales with good psychometric properties. We used this approach because there are no validated scales of irritability in existing well-established cohorts with long-term follow-up data, because of irritability only relatively recently emerging as an area of research interest (ie, the scales were developed after the conception of these cohorts). Existing longitudinal studies examining irritability in relation to longer-term outcomes use measures that are based on items from existing questionnaires such as the Child and Adolescent Psychiatric Assessment (CAPA) or the (Kiddie Schedule for Affective Disorders and Schizophrenia (K-SADS). The psychometric properties of our measure were assessed using the Cronbach α and showed moderate internal consistency (at age 3 years, α = 0.64; at 5 years, α = 0.68; and at 7 years, α = 0.69). Although moderate, these α coefficients are similar to those found for measures used in previous studies.^40^ We found higher internal consistency when using a 3-item version of the scale (excluding the item “gets over being upset quickly”), but chose to use the 4-item version for our main analyses for conceptual reasons. We repeated the main analyses using the 3-item version as a sensitivity analysis.

### Confounders

Confounders were selected based on their potential association with childhood irritability and depressive symptoms. Factors that might be on the causal pathway (ie, occurring after age 3 years, such as bullying) were not included.

We adjusted analyses for child sex and ethnicity and the following family factors: maternal age, maternal education, maternal social class, Organisation for Economic Cooperation and Development equivalized weekly family income, housing tenure, and maternal depressive symptoms. These maternal variables were selected on the basis of them being markers of socioeconomic status and/or maternal mental health. Paternal factors were not included in the main analyses, as mothers were the primary respondents in the study, and inclusion of paternal data resulted in large amounts of missing data; however, we examined the inclusion of paternal covariates presented in Table 1 in sensitivity analyses. We also adjusted for child developmental ability at age 3 years (using the Bracken School Readiness Scale [BSRS] and the British Ability Scale–vocabulary subscale [BAS-v]) and childhood internalizing and externalizing problems (measured by the SDQ with the item contributing to the irritability measure removed). Child school readiness and vocabulary score were included as markers of child developmental level, because children with developmental delays may be more at risk for both irritability and depression.^41^

**Table 1:** Sample Characteristics

Variable	In sample with ≥1 irritability measurement, outcome, and confounding variables	Complete case sample, N
**Exposures**		
Child irritability score at age 3 y		6,997
Mean (SD; range)	3.06 (1.95; 0-8)	
Child irritability score at age 5 y		6,773
Mean (SD; range)	2.31 (1.92; 0-8)	
Child irritability score at age 7 y		6,664
Mean (SD; range)	2.30 (1.94; 0-8)	
**Confounding variables**		
Child sex		7,225
Male, n (%)	3,521 (48.7)	
Female, n (%)	3,704 (51.3)	
Child ethnicity		7,225
White, n (%)	6,614 (91.5)	
Black^a^, n (%)	132 (1.8)	
South Asian^b^, n (%)	267 (3.7)	
Mixed, n (%)	176 (2.4)	
Other ethnicity, n (%)	36 (0.5)	
Maternal age at delivery, y		7,225
Mean (SD; range)	30.3 (5.55; 16-48)	
Maternal education		7,225
Non-compulsory, n (%)	3,258 (45.1)	
Compulsory only, n (%)	3,967 (54.9)	
Maternal social class		7,225
Non-manual, n (%)	4,452 (61.6)	
Manual, n (%)	2,773 (38.4)	
UK income quintile		7,225
First, n (%) lowest	1,012 (14.0)	
Second, n (%)	1,349 (18.7)	
Third, n (%)	1,519 (21.0)	
Fourth, n (%)	1,683 (23.3)	
Fifth, n (%) highest	1,662 (23.0)	
Housing tenure		7,225
Own, n (%)	5,203 (72.0)	
Rented, n (%)	1,716 (23.8)	
Living rent free/other, n (%)	306 (4.2)	
Maternal depressive symptoms at child age 3 y		7,225
Mean (SD; range)	2.99 (3.42; 0-24)	
Maternal lifetime depression before delivery		7,225
No (%)	5,517 (76.4)	
Yes (%)	1,708 (23.6)	
Paternal social class		5,914
Non-manual, n (%)	3,796 (64.2)	
Manual, n (%)	2,118 (35.8)	
Paternal depressive symptoms at child age 3 y		5,114
Mean (SD; range)	2.72 (2.91; 0-24)	
Child Bracken school readiness standardized score age 3 y		7,225
Mean (SD; range)	106.9 (15.3; 56-149)	
Child British ability score – vocabulary scale t score age 3 y		7,225
Mean (SD; range)	51.8 (10.5; 20-80)	
Child SDQ emotional symptoms subscale score age 3 y		7,225
Mean (SD; range)	1.21 (1.33; 0-10)	
Child SDQ peer problems subscale score age 3 y		7,225
Mean (SD; range)	1.38 (1.49; 0-10)	
Child SDQ hyperactivity subscale scores age 3 y		7,225
Mean (SD; range)	3.64 (2.26; 0-10)	
Child SDQ conduct problems score (temper tantrums removed) age 3 y		7,225
Mean (SD; range)	1.77 (1.50; 0-8)	
**Outcomes**		
Child depressive symptoms age 14 y		7,225
Mean (SD; range)	5.62 (5.92; 0-26)	
Child self-harm age 14 years		7,225
No (%)	6,118 (84.7)	
Yes (%)	1,107 (15.3)	

Irritability is associated cross-sectionally with internalizing and externalizing disorders, which is why we adjusted for comorbid internalizing and externalizing symptoms at baseline.^16^ Confounders were measured at the same time as the first measurement of irritability (ie, at 3 years of age). This results in difficulties disentangling confounding from potential mediation when irritability at 3 years of age or irritability slope was the exposure. We therefore entered confounders incrementally so we could inspect their influence on associations (Supplement 1, available online for further details of confounding variables).

### Statistical Analysis

All analyses were conducted in Stata16MP. First, we investigated the association between each individual irritability measurement as the exposure (ie, irritability at ages 3, 5, and 7 years) and depressive symptoms and self-harm at age 14 years as outcomes, using univariable and multivariable linear (for depressive symptoms) and logistic (for self-harm) regressions. For each exposure and outcome, we ran univariable models and then a series of multivariable models, adjusting for confounders incrementally. We adjusted for child sex and ethnicity, then added family factors and then child developmental ability at age 3 years. Finally, we included childhood internalizing and externalizing problems based on the SDQ; we included the score from the previous wave or at the same wave when a previous score was not available (ie at age 3 years and when using the slope as exposure).

We then investigated associations between the change in irritability score between ages 3 and 7 years, and depressive symptoms and self-harm at age 14 years. To create the early childhood changes in the irritability exposure variable, we fitted multi-level models to within-child repeated irritability scores at ages 3, 5, and 7 years as a function of linear and quadratic indicators of child age (Table S1, available online). These models also included a random intercept for child and random slope for time. Using this model, we predicted each child’s change in irritability score between age 3 and 7 years (ie, the within-child slope), which we standardized to have a mean of 0 and an SD of 1, and their predicted irritability score at 3 years (ie, the intercept). We used the predicted intercept as a covariate in sensitivity analyses because of it being based on the same model as the slope. However, we did not use it as an exposure in the main analyses because the the availability of data directly measuring irritability at age 3 years (Supplement 1, available online). We investigated the association between change in early childhood irritability (ie, the slope) as the exposure, and depressive symptoms and self-harm at age 14 years as outcomes using univariable and multivariable linear (depressive symptoms) and logistic (self-harm) regression. These analyses were adjusted for the same confounders and covariates described above, in a stepwise manner.

For both analyses, we examined sex differences in the association between childhood irritability and adolescent depressive symptoms or self-harm by fitting an interaction between sex and each childhood irritability exposure. We conducted analyses stratified by sex if there was evidence of an interaction. Where there is no evidence of an interaction, we have not presented the results of analyses stratified by sex because of there being no statistical evidence to support different results in male and female participants. As sensitivity analyses, we also adjusted for prior irritability; in analyses investigating irritability at age 5 and 7 years, this was done by adjusting for prior irritability. For the analyses using change in irritability as exposure, this was done by adjusting for the child’s predicted irritability intercept at 3 years to ensure that samples were comparable, but we conducted a further sensitivity analysis adjusting for the actual irritability measurement at age 3 years. In further sensitivity analyses. we included paternal social class and paternal depressive symptoms as confounders because paternal data were limited and restricted the sample size. To approximate depression potentially meeting clinical diagnostic criteria, we used the recommended cut-off on the sMFQ of ≥12^32^ and conducted sensitivity analyses with this binary variable.

### Missing Data

All analyses were conducted using the relevant MCS population weights, to account for sampling design. Missing data were assumed to be associated with observed data (ie, missing at random).^42^ To account for missing data, we conducted sensitivity analyses using multiple imputation with chained equations (MICE). We imputed missing confounder and outcome data for adolescents with data available on the exposure. We imputed 50 datasets using all variables in the main models as well as auxiliary variables (Supplement 1, available online).

## Results

### Sample and Missing Data

**Figure 1:**
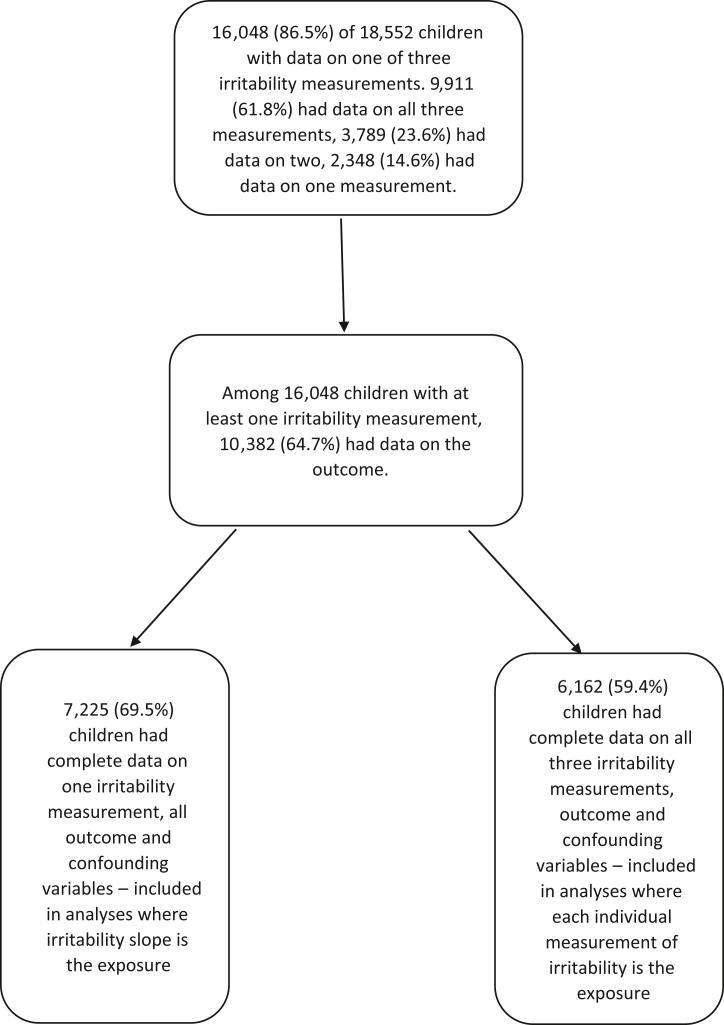
Participant Flowchart

Of the initial 18,552 children, 16,048 (86.5%) had data available on at least 1 of 3 irritability measurements. Among the children with at least 1 irritability measure, 10,382 (64.7%) had data available on the outcome. Of these, 7,225 (69.5%) had complete data on at least 1 irritability measurement and all outcome and confounding variables, and 6,162 (59.4%) had data on all 3 irritability measurements, outcome, and confounding variables (Figure 1). In the sample with complete data on at least 1 irritability measurement and all outcome and confounding variables, 51% were female and 92% were of White ethnicity. Missing data were more common among participants who were male and from ethnic minority backgrounds (Black, South Asian, Mixed, or Other) (Table 1 and Table S2, available online). Black participants were those from Black Caribbean, Black African, or other Black backgrounds. South Asian participants were those from Indian, Pakistani, or Bangladeshi backgrounds.

### Childhood Irritability

Boys, children with more depressed mothers or less educated parents, and those from more deprived and ethnic minority backgrounds had higher irritability scores (Table S3, available online). Children were more irritable at 3 years; this decreased (age coefficient = −0.17, 95% CI = −0.18 to −0.16, *p* < .001) and stabilized (age-squared coefficient = 0.09, 95% CI = 0.08-0.09, *p* < .001) over time (Table S1, available online). The change in irritability scores between age 3 and 7 years was standardized to have a mean of 0 and an SD of 1; in the sample with complete data on change in irritability, confounders, and outcome (n = 7,225), the mean change in irritability score was −0.05 (SD = 1.08; range = −4.00 to 4.81).

### Depressive Symptoms and Self-Harm at Age 14 Years

The mean sMFQ score at 14 years in those with at least 1 irritability measurement was 5.55 (SD = 5.87; range = 0-26). sMFQ scores were lower in boys (mean = 4.05, SD = 4.58, range = 0-26) than in girls (mean = 7.02, SD = 6.57, range = 0-26). In all, 14.8% of 14-year-olds with at least 1 irritability measurement reported having self-harmed. Boys reported less self-harm (8.1%) than girls (21.4%).

### Analyses Using Each Individual Measurement of Irritability as the Exposure and Depressive Symptoms and Self-Harm at 14 Years

In the univariable model, there was evidence that irritability score at age 3 years was associated with depressive symptoms (coefficient = 0.15, 95% CI = 0.06-0.24, *p* = .001) and self-harm (odds ratio [OR] = 1.06, 95% CI = 1.02-1.10, *p* = .004) at age 14 years. However, after inclusion of all confounders and covariates, this association was no longer present (Tables 2 and 3).

**Table 2:** Association Between Irritability Score at Ages 3, 5, and 7 Years and Depressive Symptoms at Age 14 Years (n = 6,162)

Model	Irritability at 3 y, coefficient (95% CI), *p*	Irritability at 5 y coefficient (95% CI), *p*	Irritability at 7 y, coefficient (95% CI), *p*
Univariable model	0.15 (0.06-0.24), *p* = .001	0.25 (0.15-0.35), *p* < 0.001	0.26 (0.18-0.35), *p* < 0.001
Adjusted model 1: child sex and child ethnicity	0.15 (0.07-0.24), *p* < .001	0.28 (0.19-0.38), *p* < .001	0.32 (0.24-0.40), *p* < .001
Adjusted model 2: model 1 plus maternal age, maternal education, maternal social class, family income quintile, family housing tenure, maternal lifetime depressive symptoms, maternal depressive symptoms at 3 y	0.07 (−0.02 to 0.16), *p* = 0.107	0.22 (0.12-0.31), *p* < .001	0.26 (0.18-0.34), *p* < .001
Adjusted model 3: model 2 plus child school readiness and vocabulary level at age 3 y	0.08 (−0.01 to 0.17), *p* = .083	0.22 (0.12-0.31), *p* < .001	0.26 (0.18-0.34), *p* < .001
Adjusted model 4: model 3 plus prior child emotional, peer, hyperactivity and conduct problems score	0.02 (−0.08 to 0.12), *p* = .742	0.20 (0.09-0.30), *p* < .001	0.21 (0.11-0.30), *p* < .001

Note: Results of univariable and multivariable linear regression models in those with complete exposure, outcome and confounder data using population weights using population weights. Depressive symptoms measured with the short Mood and Feelings Questionnaire (sMFQ) (range 0-26).

**Table 3:** Association Between Irritability Score at Ages 3, 5, and 7 Years and Self-Harm at Age 14 Years (n = 6,162)

Model	Irritability at 3 y, odds ratio (95% CI), *p*	Irritability at 5 y, odds ratio (95% CI), *p*	Irritability at 7 y, odds ratio (95% CI), *p*
Univariable model	1.06 (1.02-1.10), *p* = .004	1.08 (1.04-1.13), *p* = .001	1.09 (1.05-1.14), *p* < .001
Adjusted model 1: child sex and child ethnicity	1.07 (1.02-1.11), *p* = .002	1.10 (1.05-1.15), *p* < .001	1.12 (1.07-1.17), *p* < .001
Adjusted model 2: model 1 plus maternal age, maternal education, maternal social class, family income quintile, family housing tenure, maternal lifetime depressive symptoms, maternal depressive symptoms at 3 y	1.03 (0.99-1.08), *p* = .169	1.07 (1.02-1.12), *p* = .011	1.10 (1.05-1.15), *p* < .001
Adjusted model 3: model 2 plus child school readiness and vocabulary level at age 3 y	1.04 (0.99-1.08), *p* = .091	1.07 (1.02-1.13), *p* = .007	1.10 (1.05-1.15), *p* < .001
Adjusted model 4: model 3 plus prior child emotional, peer, hyperactivity and conduct problems score	1.02 (0.97-1.07), *p* = .410	1.06 (1.01-1.12), *p* = .037	1.08 (1.03-1.14), *p* = .003

Note: Results of univariable and multivariable linear regression models in those with complete exposure, outcome and confounder data using population weights.

Irritability at age 5 years was associated with depressive symptoms (coefficient = 0.25, 95% CI = 0.15-0.35, *p* < .001) and self-harm (OR = 1.08, 95% CI = 1.04-1.13, *p* = .001) at age 14 years in the univariable model. There remained some evidence of an association between irritability at age 5 years and depressive symptoms (coefficient = 0.20, 95% CI = 0.09-0.30, *p* < .001) and self-harm (OR = 1.06, 95% CI = 1.01-1.12, *p* = .037) after inclusion of all confounders and covariates (Tables 2 and 3).

In the univariable model, there was evidence that irritability at 7 years was associated with depressive symptoms (coefficient = 0.26, 95% CI = 0.18-0.35, *p* < .001) and self-harm (OR = 1.09, 95% CI = 1.05-1.14, *p* < .001) at 14 years. This remained the case after inclusion of all confounders and covariates (depressive symptoms coefficient = 0.21, 95% CI = 0.11-0.30, *p* < .001; self-harm OR = 1.08, 95% CI = 1.03-1.14, *p* = .003) (Tables 2 and 3). There was weak evidence of an interaction between irritability at 7 years and sex (*p* = .047) with self-harm at 14 years as the outcome; analyses stratified by sex suggested evidence of an association in boys but not in girls (Supplement 1, available online).

### Childhood Irritability Between Ages 3 and 7 Years (ie, Slope) and Depressive Symptoms and Self-Harm at Age 14 Years

In the univariable model, there was evidence that higher irritability slope scores were associated with higher depressive symptoms (coefficient = 0.22, 95% CI = 0.08-0.37, *p* = .003) and self-harm at 14 years (OR = 1.09, 95% CI = 1.01-1.16, *p* = .019). Evidence for this association remained after inclusion of all confounders and covariates (depressive symptoms coefficient = 0.31, 95% CI = 0.17-0.45, *p* < .001; self-harm OR = 1.12, 95% CI = 1.04-1.20, *p* = .002) (Table 4). There was some evidence of an interaction between irritability slope and sex (*p* = .032) where self-harm at 14 years was the outcome; analyses stratified by sex suggested evidence of an association in boys but not in girls (Supplement 1, available online).

**Table 4:** Association Between a 1-SD Increase in Irritability Slope From Age 3 to 7 Years and Depressive Symptoms and Self-Harm At Age 14 Years (n = 7,225)

Model	Depressive symptoms as a continuous outcome, coefficient (95% CI), *p*	Self-harm at 14 y (binary outcome), odds ratio (95% CI), *p*
Univariable model	0.22 (0.08-0.37), *p* = .003	1.09 (1.01-1.16), *p* = .019
Adjusted model 1: child sex and child ethnicity	0.30 (0.16-0.43), *p* < .001	1.12 (1.05-1.21), *p* = .002
Adjusted model 2: model 1 plus maternal age, maternal education, maternal social class, family income quintile, family housing tenure, maternal lifetime depressive symptoms, maternal depressive symptoms at 3 y	0.29 (0.15-0.43), *p* < .001	1.12 (1.04-1.20), *p* = .002
Adjusted model 3: model 2 plus child school readiness and vocabulary level at age 3 y	0.29 (0.15-0.43), *p* < .001	1.11 (1.04-1.19), *p* = .004
Adjusted model 4: model 3 plus child emotional, peer, hyperactivity and conduct problems score at age 3 y	0.31 (0.17-0.45), *p* < .001	1.12 (1.04-1.20), *p* = .002

Note: Results of univariable and multivariable regression models in those with complete exposure, outcome and confounder data using population weights.

### Sensitivity Analyses

Sensitivity analyses including prior irritability as an additional covariate followed a similar pattern (Tables S4a and b, available online). Results were also similar in sensitivity analyses adjusting for the predicted intercept (ie, predicted irritability at 3 years) and in analyses where irritability at 3 years was included as covariate (Table S4c, available online). Results using sMFQ scores as a binary variable (based on an sMFQ score over 12) were similar (Table S5, available online). Sensitivity analyses including paternal covariates as additional confounders followed a similar pattern (Table S6a, available online), except for analyses of the irritability slope with self-harm as the outcome. Here, there was no evidence of an association. although effect size estimates were broadly similar (Table S6b, available online). Results for sensitivity analyses conducted in samples with imputed confounder and outcome data (n = 9,911 for irritability at 3, 5, and 7 years as exposures; n = 16,048 for irritability slope as exposure) were consistent with those from complete case samples, suggesting that missing confounder and outcome data did not have a significant impact on the results (Tables S7-S9, available online). Results were also similar when using the 3-item measure of irritability (excluding the item “gets over being upset quickly”) that had higher Cronbach α (Table S10-S12, available online).

## Discussion

This is the first longitudinal population-based study of which we are aware that examines the relationship between changes in irritability during early childhood and subsequent depressive symptoms and self-harm. We found that children whose irritability did not decrease as would be expected during early childhood, and who therefore had higher irritability levels relative to other children at ages 5 and 7 years, were more likely to report depressive symptoms and self-harm at 14 years. In contrast, we found no evidence of an association with higher irritability at age 3 years and depressive symptoms or self-harm at 14 years.^25^

This study adds to the existing literature showing that irritability not only precedes depressive symptoms and self-harm but also that changes in irritability can be identified in early childhood. Given that differences in irritability emerge as early as 3 to 5 years of age, intervention during preschool-age years may be beneficial. Our study is population-based, which is important because not all children with mental health difficulties present to mental health services. This is also relevant because, although irritability is a common symptom in childhood and although work to differentiate normative and clinically significant irritability is underway, clinical cut-offs on established scales are still under development.^43^ However, in this study, whether irritability is clinically significant is not relevant to our primary aims. We aimed to study population variation in irritability in early childhood, examining the whole range of irritability, including irritability that may not be considered clinically significant, in relation to later clinically important outcomes of depression and self-harm. The implications of our findings are that irritability that does not decrease over time and that remains high at ages 5 and 7 years could increase the risk of later depression and self-harm. Therefore intervening, even in individuals with irritability that may not appear clinically significant in childhood (age ∼5-7 years), might be associated with reduced risk of depression and self-harm later in life. Irritability that does not decline by ages 5 and 7 years is therefore a potentially important symptom to address when considering public health interventions that may reduce depression and self-harm at a population level.

A number of mechanisms could explain the observed associations. Persistent irritability might increase the risk of emotion dysregulation, parenting problems, avoidance, threat perception, social exclusion and negative coping strategies. It is also possible that irritability is a reflection of an underlying susceptibility to subsequent low mood, namely, that in some individuals, childhood irritability and later depressive symptoms are different manifestations of the same underlying susceptibility to low mood. Indeed, irritability is related to the concept of negative affectivity, which is associated with depression.^17^^,^^18^ In addition, it is important to consider environmental factors, such as childhood maltreatment and bullying, that may precipitate or perpetuate irritability or prevent the development of appropriate frustration management skills.^14^^,^^18^

We found some evidence that childhood irritability was associated with self-harm among boys but not among girls; however, the reasons for this finding are unclear. This potential gender difference differs from that identified by a previous study investigating late childhood irritability and late adolescent suicidality.^26^ This may be due to the differences in timepoint that both irritability and self-harm were measured.^44^^,^^45^ Irritability levels were higher in boys than in girls in our study. Fewer girls at the more severe end of the irritability measure might have reduced our ability to detect associations. Measurement invariance is also a possibility, and irritability could be expressed differently in boys than in girls. It is also possible that higher levels of impulsivity (which is associated with irritability) in boys than in girls contributed to this gender difference. The potential sex differences that we observed would benefit from exploration using more detailed measures of self-harm and suicidality across multiple developmental stages.

The strengths of this study include the longitudinal design, large sample size, long-term follow-up, and broad range of confounders. Attrition is a limitation of this study that may have introduced selection bias; however, we used multiple imputation to investigate the impact of missing data, and this did not alter our findings. We replaced missing data in outcomes and confounders (but not exposures). Our imputed sample may have differed from the 18,552 families initially recruited. However, sample and population weights should have reduced these differences. There is also evidence that within-cohort associations remain valid even when missing data are systematic.^46^ This study is based on the UK population in a sample designed to reflect the diversity of this population; however, participants from deprived or ethnic minority backgrounds were more likely to have missing data. We attempted to account for this by adjusting for ethnicity and other sociodemographic factors, as well as multiple imputation of missing data. In addition, although this study is based on the UK population, we hope that it will stimulate international research and cross-cultural comparisons. As our findings were based on observational data, we cannot be certain that associations are causal. Residual confounding is a possibility that cannot be ruled out. For example, genetic confounding is important to consider, given the associations between irritability and depression.^47^ There were higher levels of missing data from fathers. However, adjusting for paternal depressive symptoms in sensitivity analyses had little effect on the association; polygenic risk scores were not available, but these only weakly predict risk of depression at present.^48^^,^^49^ In addition, we were not able to adjust for potential environmental confounders such as childhood maltreatment, parenting style, or bullying. Adolescent depression was measured by self-report; however, the sMFQ is a widely used measure that has been validated against longer assessments^31^, ^32^ and shown to be a valid instrument to capture latent depression traits in adolescence.^31^, ^32^ In addition, random measurement error would be more likely to reduce the effect size than to produce spurious associations. The sMFQ was based on *DSM-IV* symptoms of depression, but there were no changes to the symptoms of depression from *DSM-IV* to *DSM-5.*^50^ There were minor changes to diagnostic criteria.^50^ We used a continuous score based on depressive symptoms rather than a binary classification based on clinical criteria. We would therefore not expect changes to the diagnostic criteria to affect our findings. Our outcome assessments covered a limited time period, and studies with longer follow-ups would be useful. Irritability was measured using single items from other questionnaires combined to create a continuous score, rather than via a specifically developed measure. This was because there are no validated scales of irritability available in existing cohorts with long-term follow-up data, because of irritability emerging as an area of research interest after the conception of these cohorts. As a result, all longitudinal studies examining irritability use measures based on items from existing questionnaires such as the CAPA or K-SADS. Although our study used items from different questionnaires, the items and overall approach were similar to those in previous studies.^40^ The internal consistency of our irritability scale was moderate. Now that research into this area has progressed, validated measures of irritability (such as the Affective Reactivity Index [ARI]) have been developed, and will hopefully be used in future longitudinal studies. There was overlap between the irritability items that we used and those included in the ARI, but there are differences in wording that could be important. The ARI is also longer and assesses degree of impairment. As the irritability assessment was maternally reported, it may also have been influenced by maternal mood. In addition, self-harm was measured via the response to a single self-reported item, which did not distinguish between NSSH and suicide attempts. There is evidence that these behaviors may have different causes.^51^ Longer measures that differentiate between NSSH and suicidality would benefit future studies. Although self-reported self-harm assessments would reduce observer bias, some adolescents may have under-reported if they did not classify their behavior as self-harm. We did not have any data on suicidality alone, or any context as to how participants may have interpreted this, although we have assumed that their responses refer to any perceived deliberate self-harm within the past year. Depression could be a mediator of the association between irritability and self-harm (and the same for self-harm as the mediator of subsequent depression). We did not investigate this in our study, but this is a direction for future research.

If the associations found by our study are causal, there is potential for intervention between the ages of 3 and 7 years for children with high irritability, as well as universal support for parents in managing irritability in preschool children. There is some evidence for specific interventions targeting irritability as a transdiagnostic symptom, with further work underway^16^^,^^52^; most such interventions focus on parent management training (PMT) and cognitive−behavioral therapy (CBT). Given that the focus of our study is early childhood, the most suitable intervention would most likely be parent management training; this could be offered both as a low-intensity universal intervention to all parents of preschool children, as well as a more targeted higher-intensity intervention for parents at children with more significant symptoms. Such interventions could be incorporated into broader support for early childhood development and may play an important preventive role in reducing the prevalence of depression. Early-years investment has been shown to improve a broad range of health and socioeconomic outcomes, and may have specific a role in preventing depression and self-harm.
