# Supplementary material for: Changes in Early Childhood Irritability and Its Association With Depressive Symptoms and Self-Harm During Adolescence in a Nationally Representative United Kingdom Birth Cohort
Source: J Am Acad Child Adolesc Psychiatry. 2024 Jan;63(1):39–51. doi: 10.1016/j.jaac.2023.05.027 (PMC11163475; doi:10.1016/j.jaac.2023.05.027)
Supplement: Supplement 1 [file mmc1.doc]

**Changes in early childhood irritability and its association with depressive symptoms, and self-harm during adolescence in a nationally representative UK birth cohort**

**Supplement 1**

Table of Contents

| Study design and participants | 3 |
| --- | --- |
| Details on irritability measure | 3 |
| Details on confounders | 5 |
| Variables included in imputation model | 6 |
| Table S1 - Unconditional multilevel models used derive the | 7 |
| Table S2 – Sample characteristics in those with missing data | 8-9 |
| Table S3 - *irritability scores according to sample characteristics* | 10-11 |
| Interaction between irritability and sex with depressive symptoms and self-harm at 14 years | 12 |
| Tables S4a, 4b and 4c– analyses including prior irritability as a covariate | 13 |
| Table S5- Results using depression as a binary variable as the outcome | 14 |
| Tables S6a and 6b- Results from analyses including paternal covariates | 15 |
| Tables S7, 8 and 9– results from analyses in imputed samples | 16-17 |
| Tables S10, 11 and 12– results from analyses using the three-item measure of irritability (excluding the item ‘gets over being upset quickly’) | 18-19 |
| Using multi-level model predicted childhood irritability at three years (i.e. intercept) | 20 |
| References | 21 |

## *Study design and participants*

Families were selected for participation in the Millennium Cohort Study (MCS) from the Department of Social Security’s Child Benefit register, using a clustered stratified sampling design in order to ensure representation of infants living in more deprived areas of the UK and of those from ethnic minority backgrounds. The initial sample recruited comprised 18, 552 families and 18,818 children between September 2000 and January 2002. Subsequently, a further 682 eligible families were recruited for MCS sweep two, bringing the total sample up to 19,244 families. MCS has collected data on cohort members and their families via questionnaires and interviews when the children were aged nine months (MCS1), three (MCS2), five (MCS3), seven (MCS4), 11 (MCS5) and 14 (MCS6) years old. This study used data from waves two to six of the study when participants were aged between three to 14 years old (see supplement for details).

For each sweep of data collection ethical approval was granted by the Multi-Centre Research Ethics Committee (MREC) and parents were asked to provide written informed consent. Children were also asked to provide informed consent to participate from the age of 11 years onwards. The full details of the MCS study, recruitment and follow-up can be found on the MCS website (<https://cls.ucl.ac.uk/cls-studies/millennium-cohort-study/>). Millennium Cohort Study data are available free of cost to researchers from the UK Data Service website (<https://www.ukdataservice.ac.uk/>).

## *Irritability measure*

Childhood irritability was measured using four questions; three from the maternally completed Child Social Behaviour questionnaire (CSBQ) as follows: “Is easily frustrated”, “Gets over being upset quickly” and “Shows wide mood swings” ^1^and one from the Strengths and Difficulties Questionnaire (SDQ): “Often has temper tantrums”.

The CSBQ and SDQ were administered at ages three, five and seven years. For both measures, mothers were asked to rate each statement with the options “not true” (coded 0), “somewhat true” (coded 1), “certainly true” (coded 2) based on their child’s behaviour over the prior six months. Responses to these four irritability items were summed to create an irritability score ranging from 0-8 at each timepoint. This was used as a continuous variable in analyses.

The psychometric properties of the 4-item measure were assessed using Cronbach’s alpha and showed internal consistency (at three years α=0.64, five years α=0.68 and at seven years α=0.69) similar to previous studies. When developing the scale, we did consider whether all four items should be included as two of the items (‘gets over being upset quickly’ and ‘shows wide mood swings’) may have been interpreted by parents as not specifically relating to irritability and rather to a broader construct of negative affectivity.

When developing the scale, we found that limiting the number of items to just ‘easily frustrated’ and ‘often has temper tantrums’ reduced Cronbach’s alpha substantially. We also found that the internal consistency of the scale was not substantially different when removing ‘shows wide mood swings’ but did find that it improved when the item ‘gets over being upset quickly’ was not included in the scale (Cronbach’s alpha 0.73-0.79, rather than 0.64-0.69 when included); however, for the main analyses we chose to include this for conceptual reasons and to increase the number of items in the scale. This is justified conceptually because some scales of irritability (particularly for younger children), for example those derived from the Preschool Age Psychiatric Assessment (PAPA) do include items relating to the time it takes to recover from temper tantrums, with tantrums or excessive temper being described as ‘shouting, crying or stamping’, which can be broadly described as being upset ^2^. In addition, exploratory factor analysis of items from a range of questionnaires in younger children have found that items such as ‘tearfulness and crying’ which are not in keeping with irritability in older children, adolescents, or adults, are found to load onto the irritability factor, rather than sadness or anhedonia in younger children ^3^. This may be because in younger children, tearfulness and crying are frequently a part of temper tantrums where anger is behaviourally manifest as more generalised distress, and such behaviours are not necessarily a reflection of sadness. Similarly, mood cycling has been linked to irritability in younger children in exploratory factor analyses ^3^, this may also be because younger children tend to more overtly switch from when positive to negative affect this is related to anger rather than sadness.

In addition, given the higher internal consistency of the three-item scale we have repeated the main analyses using the three item measure (excluding ‘gets over being upset quickly’) as a sensitivity analysis.

## *Confounders*

In order to obtained unbiased estimates of the association between irritability and depressive symptoms and self-harm at 14 years, we controlled for variables which could be hypothesised to cause both the exposure and the outcome ^4,5^; exposures which were potentially on the causal pathway between exposure and outcome were not included. Potential confounding variables were identified based on previous literature and clinical observations. Confounding variables included: child’s sex as reported by their mothers at 9 months; and ethnicity reported by child’s mother at 9 months and classified according to the Office for National Statistics categories (White and Black African/Black Caribbean/Mixed/Asian/Other); family socioeconomic status at 9 months measured using maternal highest education (compulsory or non-compulsory), property ownership (owned/rented/rent-free), family weekly income equivalised for number of family members using Organisation for Economic Co-operation and Development (OECD) criteria, and maternal social class (manual/non-manual occupation); a continuous measure of maternal age at birth of the child; a self-reported measure of whether mothers had a lifetime history of depression at 9 months (yes or no) and, self-reported maternal depressive symptoms at 3 years as measured by the Kessler-6 scale ^6^. We also adjusted for child’s cognitive development, and child emotional and behavioural difficulties; where possible we adjusted for these using a measurement taken prior to the irritability measurement (i.e., where irritability at 5 years or 7 years was the exposure), but where this was not possible (i.e., where irritability at 3 years, or irritability slope was the exposure) these measurements were taken at the same timepoint as the irritability measurement. As a result, these adjustments were included in the models as separate steps due to the difficulties in disentangling temporality and therefore potential mediation. Child’s cognitive development at three years was adjusted using language development assessed via the British Ability Scales naming vocabulary subscale (BAS-v) and school readiness as measured by the Bracken School Readiness Scale (BSRS). Trained researchers administered the BAS-v subtest, measuring child’s expressive language ability,^7^ and the BSRS, measuring child’s performance on six sub-tests relating to letters, numbers, colours, sizes, comparisons, and shapes. We used standardised t-scores for the BAS and the composite standard score of the BSRS. We adjusted for child’s emotional and behavioural difficulties at three years using subscales of the Strengths and Difficulties Questionnaire (SDQ) ^8^. The SDQ is a validated scale capturing internalising (emotional symptoms and peer problems) and externalising (conduct, hyperactivity/inattention) problems via 20 questions scored on a three-point Likert scale (range 0 “not true” to 2 “certainly true”). We used the emotional symptoms, peer problems and hyperactivity/inattention subscales in their entirety and, the conduct problems subscale with the one item on temper tantrums removed, due this item being included in the irritability measure. In sensitivity analyses we also included additional paternal confounding variables. These included paternal social class (manual/non-manual) and paternal depressive symptoms at 3 years as measured by the Kessler-6 scale ^6^.

## *Variables included in imputation model*

Auxiliary variables are variables within the original data that are not included in the analysis, but which are thought to be correlated with missing variables of interest, particularly the outcome variables, or which are associated with missingness. They are not variables of interest in the analytic model but are added to the imputation model to improve power and the plausibility of the missing at random (MAR) assumption. In general, the information gained by including auxiliary variables is greater than the noise introduced by potentially irrelevant information, so inclusive strategies are recommended ^9,10^. In addition to the variables included in our main models, in imputation models we also included the following auxiliary variables: maternal smoking, maternal marital states, child internalising and externalising problems at ages three five, seven, 11 and 14 years, self-regulation at three, five and seven years, child social media use at 14 years, child social support at 14 years, child sexual orientation reported at 14 years and stratum and population weight variables.

## Table S1 - Unconditional multilevel models used derive the exposures (n=16,048)

|  | **Models with age centred at 3 years**  **(used to predict the random intercept)** | | **Models with age centred at 5 years**  **(used to predict the linear random slope)** | |
| --- | --- | --- | --- | --- |
|  | **Unconditional model 1** | **Unconditional model 2** | **Unconditional model 1** | **Unconditional model 2** |
| **Fixed effects** |  |  |  |  |
| *Age* | -0.18 (-0.18 to -0.17)  p<0.001 | -0.51 (-0.54 to -0.49)  p<0.001 | -0.18 (-0.18 to -0.17)  p<0.001 | -0.17 (-0.18 to -0.16)  p<0.001 |
| *Age^2^* | - | 0.09 (0.08 to 0.09)  p<0.001 | - | 0.09 (0.08 to 0.09)  p<0.001 |
| *Constant* | 3.16 (3.13 to 3.20) | 3.28 (3.24 to 3.31) | 2.81 (2.79 to 2.84) | 2.59 2.56 to 2.62) |
| **Random effects** |  |  |  |  |
| *SD (age)* | 0.26 (0.25 to 0.28) | 0.28 (0.26 to 0.30) | 0.26 (0.25 to 0.28) | 0.28 (0.26 to 0.30) |
| *SD (constant)* | 1.53 (1.50 to 1.56) | 1.55 (1.52 to 1.58) | 1.50 (1.48 to 1.52) | 1.51 (1.49 to 1.53) |
| *correlation (age, constant)* | -0.23 (-0.27 to -0.18) | -0.25 (-0.29 to -0.21) | 0.11 (0.08 to 0.16) | 0.11 (0.07 to 0.15) |
| *AIC* | 158149.4 | 157539.7 | 158149.4 | 157539.7 |
| *BIC* | 158200.9 | 157599.8 | 158200.9 | 157599.8 |

Fixed effects of these models show that emotion regulation scores decline overall between age 3 and 5 years and are relatively stable thereafter. Random effects coefficients indicate that there is greater variability between children in intercept values than there is in slope values.

## Table S2 – Sample characteristics among those with missing data

| **Variable**  (N in sample with complete exposure measures; N in complete case sample; N with missing data) | **In sample with at least one irritability measurement** | **In those with missing data** |
| --- | --- | --- |
| **Exposures** |  |  |
| **Child irritability score at age three years** (13450; 6997; 6435)  *mean (sd; range)* | 3.25 (1.98; 0-8) | 3.44 (2.00; 0-8) |
| **Child irritability score at age five years** (13658; 6773; 6885)  *mean (sd; range)* | 2.57 (2.00; 0-8) | 2.83 (2.04; 0-8) |
| **Child irritability score at age seven years** (12551; 6664; 5887)  *mean (sd; range)* | 2.56 (2.02; 0-8) | 2.85 (2.06; 0-8) |
| **Confounding variables** |  |  |
| **Child sex**  (16048; 7225; 8823)  Male, n (%)  Female, n (%) | 8173 (50.9)  7875 (49.1) | 4652 (52.7)  4171 (47.3) |
| **Child ethnicity**  (16048; 7225; 8823)  White, n (%)  Black^a^, n (%)  South Asian^b^, n (%)  Mixed, n (%)  Other ethnicity, n (%) | 13530 (84.3)  526 (3.3)  1332 (8.3)  466 (2.9)  194 (1.2) | 6916 (78.4)  394 (4.5)  1065 (12.1)  290 (3.3)  158 (1.8) |
| **Maternal age at delivery, years**  (16010; 7225; 8785)  *mean (sd; range)* | 29.3 (5.9; 14-52) | 28.5 (6.09; 14-52) |
| **Maternal education**  (15986; 7225; 8761)  Non-compulsory, n (%)  Compulsory only, n (%) | 5648 (35.3)  10338 (64.7) | 2390 (27.3)  6371 (72.7) |
| **Maternal social class**  (14397; 7225; 7172)  Non-manual, n (%)  Manual, n (%) | 7745 (53.9)  6652 (46.2) | 3293 (45.9)  3879 (54.1) |
| **UK income quintile**  (16003; 7225; 8778)  1^st^, n (%) lowest  2^nd^, n (%)  3^rd^, n (%)  4^th^, n (%)  5^th^, n (%) highest | 3765 (23.5)  3510 (21.9)  3082 (19.2)  2944 (18.4)  2701 (16.9) | 2753 (31.4)  2161 (24.6)  1563 (17.8)  1261 (14.4)  1040 (11.8) |
| **Housing tenure**  (16018; 7225; 8793)  Own, n (%)  Rented, n (%)  Living rent free/other, n(%) | 9651 (60.2)  5389 (33.7)  969 (6.1) | 4448 (50.6)  3682 (41.9)  663 (7.5) |
| **Maternal depressive symptoms at child age three years (**12956; 7225; 5731)  *mean (sd; range)* | 3.25 (3.74; 0-24) | 3.57 (4.10; 0-24) |
| **Maternal lifetime depression before delivery** (16034; 7225; 8809)  No (%)  Yes (%) | 12036 (75.1)  3998 (24.9) | 6519 (74.0)  2290 (26.0) |
| **Paternal social class**  (11692; 5914; 5778)  Non-manual, n (%)  Manual, n (%) | 6689 (57.2)  5003 (42.8) | 2893 (50.1)  2885 (49.9) |
| **Paternal depressive symptoms at child age three years** (8964; 5114; 3850)  *mean (sd; range)* | 2.82 (3.09; 0-24) | 2.95 (3.31; 0-24) |
| **Child Bracken school readiness standardised score age three years** 13045; 7225; 5820)  *mean (sd; range)* | 103.8 (16.2; 56-149) | 100.0 (16.5; 56-146) |
| **Child British ability score – vocabulary scale t-score age three years** (13715; 7225; 6490)  *mean (sd; range)* | 49.7 (11.3; 20-80) | 47.3 (11.7; 20-80) |
| **Child SDQ emotional symptoms subscale score age three years**  (14010; 7225; 6785)  *mean (sd; range)* | 1.37 (1.49; 0-10) | 1.54 (1.65; 0-10) |
| **Child SDQ peer problems subscale score age three years**  (13921; 7225; 6696)  *mean (sd; range)* | 1.55 (1.59; 0-10) | 1.73 (1.67; 0-10) |
| **Child SDQ hyperactivity subscale scores age three years**  (13914; 7225; 6689)  *mean (sd; range)* | 3.90 (2.36; 0-10) | 4.19 (2.44; 0-10) |
| **Child SDQ conduct problems score (temper tantrums removed) age three years** (13925; 7225; 6700)  *mean (sd; range)* | 1.93 (1.60; 0-8) | 2.10 (1.69; 0-8) |
| **Outcomes** |  |  |
| **Child depressive symptoms age 14 years** (10382; 7225; 3157)  *mean (sd; range)* | 5.55 (5.86; 0-26) | 5.38 (5.73; 0-26) |
| **Child self-harm age 14 years**  (10474; 7225; 3249)  No (%)  Yes (%) | 8924 (85.2)  1550 (14.8) | 2806 (86.4)  443 (13.6) |

## *Table S3 – irritability scores according to sample characteristics*

| **Variable**  (N for complete case sample with all three irritability measures; N for complete case sample with at least one irritability measure) | **Irritability at each timepoint (n=6162, n=4212 for paternal data)** | | | **Irritability slope (n=7225, n=4706 for paternal data)** |
| --- | --- | --- | --- | --- |
|  | **Three years** | **Five years** | **Seven years** |  |
| **Child sex**  Male (2990; 3521)  Female (3172; 3704) | 3.07 (1.97)  3.00 (1.93) | 2.39 (1.93)  2.20 (1.88) | 2.43 (2.01)  2.15 (1.87) | -0.17 (0.15)  -0.19 (0.14) |
| **Child ethnicity**  White (5734; 6614)  Ethnic minority (428; 611) | 3.02 (1.94)  3.16 (2.04) | 2.27 (1.90)  2.59 (1.97) | 2.27 (1.94)  2.42 (2.00) | -0.18 (0.15)  -0.18 (0.15) |
| **Maternal age at delivery, years**  14-19 (159; 221)  20-29 (2238;2761)  30-29 (3505; 3954)  >40 (260; 260) | 3.97 (1.80)  3.35 (1.98)  2.82 (1.90)  2.50 (1.96) | 3.22 (2.16)  2.62 (1.95)  2.07 (1.84)  1.77 (1.70) | 3.41 (2.22)  2.59 (2.02)  2.08 (1.85)  1.73 (1.75) | -0.16 (0.16)  -0.18 (0.15)  -0.18 (0.14)  -0.19 (0.15) |
| **Maternal education**  Non-compulsory (2947; 3258)  Compulsory only (3215; 3967) | 2.70 (1.87)  3.33 (1.97) | 1.98 (1.80)  2.57 (1.96) | 2.01 (1.84)  2.53 (2.00) | -0.18 (0.14)  -0.18 (0.15) |
| **Maternal social class**  Non-manual (3981; 4452)  Manual (2181; 2773) | 2.76 (1.89)  3.51 (1.96) | 2.06 (1.80)  2.71 (2.03) | 2.06 (1.85)  2.68 (2.04) | -0.18 (0.14)  -0.18 (0.15) |
| **UK income quintile**  1^st^ lowest (734; 1012)  2^nd^ (1092; 1349)  3^rd^ (1296; 1519)  4^th^ (1518; 1683)  5^th^ highest (1522; 1662) | 3.66 (1.96)  3.46 (2.00)  3.10 (1.91)  2.78 (1.87)  2.61 (1.88) | 3.02 (2.08)  2.68 (2.03)  2.29 (1.89)  2.05 (1.78)  1.90 (1.72) | 2.92 (2.08)  2.61 (2.05)  2.31 (1.96)  2.07 (1.84)  1.93 (1.77) | -0.18 (0.15)  -0.18 (0.15)  -0.18 (0.15)  -0.18 (0.14)  -0.18 (0.14) |
| **Housing tenure**  Own (4619; 5203)  Rented (1308; 1716)  Living rent free/other (235; 306) | 2.86 (1.90)  3.61 (2.01)  3.25 (1.97) | 2.10 (1.82)  2.95 (2.07)  2.41 (1.91) | 2.10 (1.86)  2.92 (2.11)  2.38 (2.96) | -0.18 (0.14)  -0.17 (0.15)  -0.19 (0.15) |
| **Thirds of maternal depressive symptoms at child age three years**  Lowest (1517; 1775)  Middle (2778; 3194)  High (1867; 2256) | 2.57 (1.87)  2.89 (1.89)  3.61 (1.97) | 1.78 (1.72)  2.18 (1.82)  2.87 (2.04) | 1.80 (1.77)  2.22 (1.89)  2.77 (2.05) | -0.19 (0.14)  -0.18 (0.14)  -0.18 (0.15) |
| **Maternal lifetime depression before delivery**  No (4725; 5517)  Yes (1437; 1708) | 2.91 (1.91)  3.44 (2.02) | 2.18 (1.86)  2.66 (2.02) | 2.16 (1.87)  2.70 (2.11) | -0.18 (0.14)  -0.17 (0.16) |
| **Paternal social class**  Non-manual (3432; 3796)  Manual (1759; 2118) | 2.78 (1.90)  3.30 (1.98) | 2.05 (1.81)  2.52 (2.00) | 2.03 (1.82)  2.56 (2.04) | -0.18 (0.14)  -0.18 (0.15) |
| **Thirds of paternal depressive symptoms at child age three years**  Lowest (1137; 1291)  Middle (1496; 1663)  Highest (1903; 2160) | 2.94 (1.93)  2.85 (1.91)  3.05 (1.97) | 2.11 (1.86)  2.08 (1.81)  2.34 (1.91) | 2.03 (1.85)  2.13 (1.84)  2.38 (1.96) | -0.19 (0.14)  -0.18 (0.14)  -0.17 (0.15) |
| **Thirds of child Bracken school readiness standardised score age three years**  Lowest (1370; 1746)  Middle (2132; 2487)  Highest (2660; 2992) | 3.53 (1.97)  3.15 (1.95)  2.68 (1.87) | 2.72 (2.05)  2.36 (1.91)  2.00 (1.78) | 2.67 (2.06)  2.35 (1.95)  2.03 (1.84) | -0.18 (0.15)  -0.18 (0.14)  -0.18 (0.14) |
| **Thirds of child British ability score – vocabulary scale t-score age three years**  Lowest (1262; 1591)  Middle (2115; 2512)  Highest (2785; 3122) | 3.47 (1.99)  3.08 (1.96)  2.79 (1.89) | 2.71 (2.04)  2.38 (1.94)  2.03 (1.78) | 2.64 (1.99)  2.35 (2.00)  2.07 (1.86) | -0.18 (0.16)  -0.18 (0.14)  -0.18 (0.14) |
| **Child SDQ emotional symptoms subscale score age three years**  Lowest (2273; 2637)  Middle (1995; 2313)  Highest (1894; 2275) | 2..56 (1.83)  2.93 (1.90)  3.70 (1.97) | 1.91 (1.76)  2.22 (1.86)  2.82 (2.01) | 1.95 (1.86)  2.21 (1.88)  2.75 (2.02) | -0.18 (0.14)  -0.18 (0.15)  -0.19 (0.15) |
| **Child SDQ peer problems subscale score age three years**  Lowest (2338; 2669)  Middle (1592;1869)  Highest (2232; 2687) | 2.59 (1.85)  3.03 (1.88)  3.49 (1.99) | 1.89 (1.73)  2.18 (1.82)  2.78 (2.04) | 1.89 (1.80)  2.23 (1.87)  2.73 (2.05) | -0.18 (0.14)  -0.18 (0.15)  -0.18 (0.15) |
| **Child SDQ hyperactivity subscale scores age three years**  Lowest (2183; 2490)  Middle (2018; 2360)  Highest (1961; 2375) | 2.17 (1.73)  2.97 (1.79)  4.04 (1.87) | 1.62 (1.63)  2.25 (1.80)  3.08 (2.01) | 1.68 (1.69)  2.21 (1.82)  3.03 (2.08 | -0.17 (0.14)  -0.18 (0.14)  -0.19 (0.15) |
| **Child SDQ conduct problems score (temper tantrums removed) age three years**  Lowest (1365;  Middle (1738;  Highest (3059; | 1.76 (1.57)  2.52 (1.73)  3.89 (1.81) | 1.35 (1.47)  1.88 (1.69)  2.94 (1.96) | 1.41 (1.52)  1.96 (1.79)  2.86 (2.02) | -0.16 (0.13)  -0.17 (0.14)  -0.19 (0.15) |
| **Child depressive symptoms age 14 years**  No (5198; 6081)  Yes (964; 1144) | 3.01 (1.94)  3.15 (2.01) | 2.24 (1.89)  2.50 (2.02) | 2.24 (1.92)  2.51 (2.06) | -0.18 (0.14)  -0.17 (0.15) |
| **Child self-harm age 14 years**  No (5218; 6118)  Yes (944; 1107) | 3.00 (1.95)  3.18 (1.95) | 2.26 (1.89)  2.47 (2.01) | 2.24 (1.92)  2.51 (2.09) | -0.18 (0.14)  -0.17 (0.15) |

## *Interaction between irritability and sex with depressive symptoms and self-harm at 14 years*

There was no evidence of an interaction between irritability at 3 years and sex in adjusted (depressive symptoms p=0.450; self-harm p=0.950) models. This was also the case for irritability at 5 years (depressive symptoms p=0.157; self-harm p=0.106). There was no evidence of an interaction between irritability at 7 years and sex with depressive symptoms as the outcome (p=0.169) but there was weak evidence of an interaction for irritability at 7 years with self-harm as the outcome (p=0.047). In analyses stratified by sex, there was evidence of an association between irritability at 7 years and self-harm for boys (n=2990, OR 1.17, 95%CI 1.07-1.27), but not girls (n=3172, OR 1.05, 95%CI 0.99-1.11).

There was no evidence of an interaction between irritability slope and sex in adjusted models with depressive symptoms at 14 years as the outcome (p=0.441). For self-harm as the outcome, there was weak evidence of an interaction between change in irritability slope, and sex in fully adjusted (p=0.032) models. In analyses stratified by sex, there was some evidence that change in irritability was more likely to be associated with self-harm during adolescence in boys but not girls (boys: n=3521, OR 1.28, 1.10-1.49; girls: n=3704, OR 1.05, 95%CI 0.97-1.14).

## *Tables S4a 4b & 4c – analyses including prior irritability as a covariate*

Supplementary table 4a – results from univariable and multivariable linear regression models examining the association between irritability score at 3, 5 and 7 years and depressive symptoms at 14 years in those with complete exposure, outcome and confounder data (n=6162 at 3, 5 and 7 years) using population weights

| **Model** | **Increase in depressive symptoms as a continuous outcome (n=7225)**  **Coef (95% CI), p-value** | **Self-harm at 14 years (binary outcome) (n=7225)**  **Odds ratio (95% CI), p-value** |
| --- | --- | --- |
| Univariable model | 0.22 (0.08-0.37), p=0.003 | 1.09 (1.01-1.16), p=0.019 |
| Adjusted model 5: model 4 plus predicted irritability intercept at 3 years | 0.31 (0.18-0.45), p<0.001 | 1.12 (1.05-1.20), p=0.002 |
| Adjusted model 6: model 4 plus irritability at 3 years (n=6997) | 0.40 (0.24-0.55), p<0.001 | 1.15 (1.06-1.25), p=0.001 |

| **Model** | **Irritability at 3 years (n=6162)**  **Coef. (95% CI), p-value** | **Irritability at 5 years (n=6162)**  **Coef. (95% CI), p-value** | **Irritability at 7 years (n=6162)**  **Coef. (95% CI), p-value** |
| --- | --- | --- | --- |
| Univariable model | 0.15 (0.06-0.24), p=0.001 | 0.25 (0.15-0.35), p<0.001 | 0.26 (0.18-0.35), p<0.001 |
| Adjusted model 5: model 4 plus irritability at 3 years | -- | 0.22 (0.10-0.34), p<0.001 | 0.23 (0.13-0.32), p<0.001 |
| Adjusted model 6: model 5 plus irritability at 5 years | -- | -- | 0.19 (0.09-0.29), p<0.001 |

Supplementary table 4b – results from univariable and multivariable linear regression models examining the association between irritability score at 3, 5 and 7 years and self-harm at 14 years in those with complete exposure, outcome and confounder data (n=6162 at 3, 5 and 7 years) using population weights

| **Model** | **Irritability at 3 years (n=6162)**  **Odds ratio (95% CI), p-value** | **Irritability at 5 years (n=6162)**  **Odds ratio (95% CI), p-value** | **Irritability at 7 years (n=6162)**  **Odds ratio (95% CI), p-value** |
| --- | --- | --- | --- |
| Univariable model | 1.06 (1.02-1.10), p=0.004 | 1.08 (1.04-1.13), p=0.001 | 1.09 (1.05-1.14), p<0.001 |
| Adjusted model 5: model 4 plus irritability at 3 years | -- | 1.06 (1.00-1.13), p=0.057 | 1.09 (1.03-1.14), p=0.002 |
| Adjusted model 6: model 5 plus irritability at 5 years | -- | -- | 1.08 (1.02-1.14), p=0.007 |

Supplementary table 4c – results from univariable and multivariable linear regression models examining the association between increase in irritability slope from 3 to 7 years with depressive symptoms and self-harm at 14 years in those with complete exposure, outcome and confounder data (n=7225) using population weights

## *Table S5 - Results using depression as a binary variable as the outcome*

| **Model** | **Irritability at 3 years (n=6162)**  **Odds ratio (95% CI), p-value** | **Irritability at 5 years (n=6162)**  **Odds ratio (95% CI), p-value** | **Irritability at 7 years (n=6162)**  **Odds ratio (95% CI), p-value** | **Irritability slope (7225)**  **Odds ratio (95% CI), p-value** |
| --- | --- | --- | --- | --- |
| Univariable model | 1.05 (1.01-1.10), p=0.021 | 1.10 (1.05-1.15), p<0.001 | 1.09 (1.05-1.14), p<0.001 | 1.10 (1.04-1.17), p=0.002 |
| Adjusted model 1: child sex and child ethnicity | 1.06 (1.01-1.11), p=0.012 | 1.12 (1.07-1.16), p<0.001 | 1.11 (1.08-1.17), p<0.001 | 1.14 (1.07-1.21), p<0.001 |
| Adjusted model 2: model 1 plus maternal age, maternal education, maternal social class, family income quintile, family housing tenure, maternal lifetime depressive symptoms, maternal depressive symptoms at 3 years | 1.02 (0.97-1.07), p=0.430 | 1.08 (1.04-1.13), p<0.001 | 1.09 (1.05-1.14), p<0.001 | 1.13 (1.06-1.20), p<0.001 |
| Adjusted model 3: model 2 plus child school readiness and vocabulary level at age 3 years | 1.03 (0.98-1.08), p=0.294 | 1.09 (1.04-1.13), p<0.001 | 1.10 (1.05-1.14), p<0.001 | 1.12 (1.06-1.19), p<0.001 |
| Adjusted model 4: model 3 plus prior child emotional, peer, hyperactivity and conduct problems score | 1.00 (0.95-1.05), p=0.903 | 1.08 (1.03-1.13), p=0.003 | 1.07 (1.03-1.12), p=0.002 | 1.13 (1.07-1.20), p<0.001 |

Supplementary table 5 – results from univariable and multivariable linear regression models examining the association between irritability score at 3, 5 and 7 years, and increase in irritability slope from 3 to 7 years with, depressive symptoms as a binary variable at 14 years in those with complete exposure, outcome and confounder data (n=6162 at 3, 5 and 7 years, n=7225 for irritability slope) using population weights

## *Supplementary tables 6a and 6b - Results from analyses including paternal covariates*

Supplementary table 6a - Results of univariable and multivariable regression models examining the association between irritability score at 3, 5 and 7 years respectively with depressive symptoms and self-harm at 14 years in a sample with complete exposure, outcome and confounder data, including paternal covariates (n=4212) and in a sample with imputed outcome and confounder data, including paternal covariates (n=9911)

| **Model** | **Irritability at 3 years**  **Odds ratio (95% CI), p-value** | **Irritability at 5 years**  **Odds ratio (95% CI), p-value** | **Irritability at 7 years**  **Odds ratio (95% CI), p-value** |
| --- | --- | --- | --- |
| **Outcome: depressive symptoms (continuous)^$^**  Univariable | 0.15 (0.05-0.26), p=0.005 | 0.26 (0.15-0.37), p<0.001 | 0.23 (0.13-0.35), p<0.001 |
| Fully adjusted* | 0.03 (-0.09-0.15), p=0.602 | 0.21 (0.10-0.33), p<0.001 | 0.20 (0.08-0.32), p<0.001 |
| Fully adjusted* + irritability at 3 years | -- | 0.24 (0.11-0.37), p<0.001 | 0.21 (0.08-0.33), p=0.001 |
| Fully adjusted* + irritability at 5 years | -- | -- | 0.15 (0.02-0.028), p=0.025 |
| Fully adjusted* in imputed sample (n=9911) | 0.01 (-0.08-0.10), p=0.817 | 0.17 (0.08-0.26), p<0.001 | 0.18 (0.10-0.27), p<0.001 |
| Fully adjusted* + irritability at 3 in imputed sample (n=9911) | -- | 0.19 (0.10-0.29), p<0.001 | 0.20 (0.11-0.29), p<0.001 |
| Fully adjusted* + irritability at 5 in imputed sample (n=9911) | -- | -- | 0.18 (0.08-0.28), p=0.001 |
| **Outcome: self-harm (binary)^$^**  Univariable | 1.07 (1.02-1.13), p=0.004 | 1.09 (1.04-1.15), p=0.001 | 1.09 (1.03-1.15), p=0.002 |
| Fully adjusted* | 1.04 (0.97-1.10), p=0.253 | 1.07 (1.02-1.14), p=0.034 | 1.09 (1.02-1.16), p=0.013 |
| Fully adjusted* + irritability at 3 years | -- | 1.07 (0.99-1.15), p=0.082 | 1.08 (1.03-1.14), p=0.002 |
| Fully adjusted* + irritability at 5 years | -- | -- | 1.08 (1.02-1.14), p=0.007 |
| Fully adjusted* in imputed sample (n=9911) | 1.03 (0.98-1.07), p=0.295 | 1.06 (1.01-1.11), p=0.013 | 1.08 (1.03-1.13), p=0.001 |
| Fully adjusted* + irritability at 3 in imputed sample (n=9911) | -- | 1.06 (1.01-1.12), p=0.024 | 1.08 (1.03-1.13), p=0.001 |
| Fully adjusted* + irritability at 5 in imputed sample (n=9911) | -- | -- | 1.08 (1.03-1.14), p=0.004 |

Supplementary table 6b - Results of univariable and multivariable regression examining the association between a 1 SD increase in irritability slope from 3-7 years and depressive symptoms and self-harm at 14 years in a sample with complete exposure, outcome, and confounder data, including paternal covariates (n=4706) and in a sample with imputed outcome and confounder data, including paternal covariates (n=16048)

| **Model** | **Depressive symptoms as a continuous outcome at age 14 years^$^**  **Coef. (95% CI), p-value** | **Self-harm (binary outcome) at 14 years^$^**  **Odds ratio (95% CI), p-value** |
| --- | --- | --- |
| Univariable | 0.16 (-0.02-0.35), p=0.077 | 1.05 (0.95-1.16), p=0.329 |
| Fully adjusted* | 0.26 (0.09-0.43), p=0.003 | 1.08 (0.98-1.19), p=0.107 |
| Fully adjusted* + intercept | 0.27 (0.10-0.44), p=0.002 | 1.08 (0.99-1.19), p=0.094 |
| Fully adjusted* + irritability at 3 years (n=4577) | 0.36 (0.17-0.55), p<0.001 | 1.13 (0.02-1.26), p=0.025 |
| Fully adjusted* imputed sample (n=16048) | 0.32 (0.19-0.44), p<0.001 | 1.11 (1.05-1.18), p<0.001 |
| Fully adjusted* + intercept in imputed sample (n=16048) | 0.32 (0.19-0.44), p<0.001 | 1.11 (1.05-1.18), p<0.001 |
| Fully adjusted* + irritability at 3 in imputed sample (n=13450) | 0.38 (0.23-0.52). p<0.001 | 1.15 (1.07-1.24), p<0.001 |

##

**Tables S7, 8 and 9 – results from analyses in imputed samples**

**Table S7. Results of univariable and multivariable linear regression models examining the association between irritability score at age three years, five years, and seven year, with depressive symptoms at 14 years, as measured by the sMFQ in those with complete exposure data and imputed outcome and confounder data using population weights (n=****9911)**

| **Model** | **Irritability at 3 years**  **Coef. (95% CI), p-value** | **Irritability at 5 years**  **Coef. (95% CI), p-value** | **Irritability at 7 years**  **Coef. (95% CI), p-value** |
| --- | --- | --- | --- |
| Univariable model | 0.14 (0.06-0.21), p=0.001 | 0.22 (0.15-0.30), p<0.001 | 0.25 (0.17-0.32), p<0.001 |
| Adjusted model 1: child sex and child ethnicity | 0.15 (0.08-0.23), p<0.001 | 0.27 (0.19-0.34), p<0.001 | 0.31 (0.24-0.38), p<0.001 |
| Adjusted model 2: model 1 plus maternal age, maternal education, maternal social class, family income quintile, family housing tenure, maternal lifetime depressive symptoms, maternal depressive symptoms at 3 years | 0.06 (-0.01-0.14), p=0.105 | 0.19 (0.11-0.27), p<0.001 | 0.24 (0.16-0.32), p<0.001 |
| Adjusted model 3: model 2 plus child school readiness and vocabulary level at age 3 years | 0.07 (-0.01-0.15), p=0.072 | 0.20 (0.12-0.28), p<0.001 | 0.25 (0.17-0.32), p<0.001 |
| Adjusted model 4: model 3 plus prior child emotional, peer, hyperactivity, and conduct problems score | 0.01 (-0.08-0.10), p=0.768 | 0.18 (0.09-0.27), p<0.001 | 0.19 (0.10-0.27), p<0.001 |
| Adjusted model 5: model 4 plus irritability at 3 years | -- | 0.20 (0.10-0.29), p<0.001 | 0.21 (0.12-0.30), p<0.001 |
| Adjusted model 6: model 5 plus irritability at 5 years | -- | -- | 0.18 (0.08-0.28), p<0.001 |

***** - Adjusted for child sex and child ethnicity maternal age, maternal education, maternal social class, family income quintile, family housing tenure, maternal lifetime depressive symptoms, maternal depressive symptoms, child school readiness and vocabulary level at age 3 years, prior child emotional, peer, hyperactivity and conduct problems score (i.e., at 3 years for irritability at 3 years, at 3 years for irritability at 5 years and at 5 years for irritability at 7 years), paternal social class and paternal depressive symptoms

**$** - Linear regression models use where depressive symptoms measured by the sMFQ used as a continuous variable, and logistic regression models used for self-harm and depression used as a binary variable

**Table S8. Results of univariable and multivariable linear regression models examining the association between irritability score at age three years, five years, and seven year, with self-harm at 14 years, as measured by the sMFQ in those with complete exposure data and imputed outcome and confounder data using population weights (n=9,911)**

| **Model** | **Irritability at 3 years**  **Odds ratio (95% CI), p-value** | **Irritability at 5 years**  **Odds ratio (95% CI), p-value** | **Irritability at 7 years**  **Odds ratio (95% CI), p-value** |
| --- | --- | --- | --- |
| Univariable model | 1.05 (1.02-1.09), p=0.004 | 1.07 (1.03-1.11), p<0.001 | 1.09 (1.05-1.13), p<0.001 |
| Adjusted model 1: child sex and child ethnicity | 1.06 (1.02-1.10), p=0.001 | 1.09 (1.05-1.13), p<0.001 | 1.12 (1.08-1.16), p<0.001 |
| Adjusted model 2: model 1 plus maternal age, maternal education, maternal social class, family income quintile, family housing tenure, maternal lifetime depressive symptoms, maternal depressive symptoms at 3 years | 1.03 (0.99-1.07), p=0.125 | 1.06 (1.02-1.11), p=0.005 | 1.09 (1.05-1.14), p<0.001 |
| Adjusted model 3: model 2 plus child school readiness and vocabulary level at age 3 years | 1.04 (1.00-1.08), p=0.058 | 1.07 (1.02-1.12), p=0.002 | 1.10 (1.05-1.14), p<0.001 |
| Adjusted model 4: model 3 plus prior child emotional, peer, hyperactivity and conduct problems score | 1.03 (0.98-1.07), p=0.284 | 1.06 (1.01-1.11), p=0.012 | 1.08 (1.03-1.13), p=0.001 |
| Adjusted model 5: model 4 plus irritability at 3 years | -- | 1.06 (1.01-0.112), p=0.022 | 1.08 (1.03-1.14), p=0.001 |
| Adjusted model 6: model 5 plus irritability at 5 years | -- | -- | 1.08 (1.03-1.14), p=0.003 |

**Table S9. Results of univariable and multivariable models examining the association between a one standard deviation increase in irritability slope from age three to seven years and depressive symptoms and self-harm at 14 years in those with complete exposure data and imputed outcome and confounder data using population weights (n=****16,048)**

| **Model** | **Depressive symptoms as a continuous outcome**  **Coef (95% CI), p-value** | **Self-harm at 14 years (binary outcome)**  **Odds ratio (95% CI), p-value** |
| --- | --- | --- |
| Univariable model | 0.26 (0.13-0.39), p<0.001 | 1.09 (1.03-1.15), p=0.005 |
| Adjusted model 1: child sex and child ethnicity | 0.33 (0.21-0.45), p<0.001 | 1.12 (1.06-1.19), p<0.001 |
| Adjusted model 2: model 1 plus maternal age, maternal education, maternal social class, family income quintile, family housing tenure, maternal lifetime depressive symptoms, maternal depressive symptoms at 3 years | 0.31 (0.18-0.43), p<0.001 | 1.11 (1.05-1.18), p<0.001 |
| Adjusted model 3: model 2 plus child school readiness and vocabulary level at age 3 years | 0.30 (0.18-0.43), p<0.001 | 1.11 (1.05-1.18), p=0.001 |
| Adjusted model 4: model 3 plus child emotional, peer, hyperactivity and conduct problems score at age 3 years | 0.32 (0.20-0.44), p<0.001 | 1.11 (1.05-1.18), p<0.001 |
| Adjusted model 5: model 4 plus irritability intercept | 0.32 (0.20-0.44), p<0.001 | 1.11 (1.05-1.18), p<0.001 |
| Adjusted model 6: model 4 plus irritability at 3 years (n=13450) | 0.39 (0.24-0.53), p<0.001 | 1.16 (1.07-1.24), p<0.001 |

## *Tables S10, 11 and 12 – results from analyses using the three-item measure of irritability (excluding the item ‘gets over being upset quickly’)*

**Table S10. Results of univariable and multivariable linear regression models examining the association between irritability score at age three years, five years, and seven years (using the three-item irritability measure which excludes the item ‘gets over being upset quickly’), with depressive symptoms at 14 years, as measured by the sMFQ in those with complete exposure, confounder and outcome data using population weights (n=6162)**

| **Model** | **Irritability at 3 years**  **Coef. (95% CI), p-value** | **Irritability at 5 years**  **Coef. (95% CI), p-value** | **Irritability at 7 years**  **Coef. (95% CI), p-value** |
| --- | --- | --- | --- |
| Univariable model | 0.16 (0.06-0.26), p=0.001 | 0.25 (0.13-0.36), p<0.001 | 0.28 (0.18-0.38), p<0.001 |
| Adjusted model 1: child sex and child ethnicity | 0.17 (0.08-0.26), p<0.001 | 0.29 (0.19-0.40), p<0.001 | 0.35 (0.26-0.44), p<0.001 |
| Adjusted model 2: model 1 plus maternal age, maternal education, maternal social class, family income quintile, family housing tenure, maternal lifetime depressive symptoms, maternal depressive symptoms at 3 years | 0.08 (-0.01-0.18), p=0.095 | 0.21 (0.10-0.32), p<0.001 | 0.28 (0.19-0.38), p<0.001 |
| Adjusted model 3: model 2 plus child school readiness and vocabulary level at age 3 years | 0.09 (-0.01-0.19), p=0.072 | 0.22 (0.11-0.32), p<0.001 | 0.29 (0.19-0.38), p<0.001 |
| Adjusted model 4: model 3 plus prior child emotional, peer, hyperactivity, and conduct problems score | 0.01 (-0.09-0.12), p=0.785 | 0.18 (0.06-0.30), p=0.004 | 0.23 (0.12-0.33), p<0.001 |
| Adjusted model 5: model 4 plus irritability at 3 years | -- | 0.20 (0.06-0.34), p=0.004 | 0.25 (0.13-0.36), p<0.001 |
| Adjusted model 6: model 5 plus irritability at 5 years | -- | -- | 0.23 (0.11-0.35), p<0.001 |

**Table S11. Results of univariable and multivariable linear regression models examining the association between irritability score at age three years, five years, and seven years (using the three-item irritability measure which excludes the item ‘gets over being upset quickly’), with self-harm at 14 years, as measured by the sMFQ in those with complete exposure, confounder and outcome data using population weights (n=6162)**

| **Model** | **Irritability at 3 years**  **Odds ratio (95% CI), p-value** | **Irritability at 5 years**  **Odds ratio (95% CI), p-value** | **Irritability at 7 years**  **Odds ratio (95% CI), p-value** |
| --- | --- | --- | --- |
| Univariable model | 1.07 (1.02-1.11), p=0.003 | 1.10 (1.05-1.15), p<0.001 | 1.10 (1.04-1.15), p<0.001 |
| Adjusted model 1: child sex and child ethnicity | 1.07 (1.03-1.12), p=0.002 | 1.12 (1.07-1.18), p<0.001 | 1.13 (1.08-1.19), p<0.001 |
| Adjusted model 2: model 1 plus maternal age, maternal education, maternal social class, family income quintile, family housing tenure, maternal lifetime depressive symptoms, maternal depressive symptoms at 3 years | 1.03 (0.99-1.08), p=0.149 | 1.09 (1.03-1.15), p=0.003 | 1.10 (1.04-1.16), p<0.001 |
| Adjusted model 3: model 2 plus child school readiness and vocabulary level at age 3 years | 1.04 (1.00-1.09), p=0.076 | 1.09 (1.03-1.15), p=0.001 | 1.10 (1.05-1.17), p<0.001 |
| Adjusted model 4: model 3 plus prior child emotional, peer, hyperactivity and conduct problems score | 1.02 (0.97-1.08), p=0.420 | 1.08 (1.02-1.15), p=0.013 | 1.08 (1.02-1.15), p=0.008 |
| Adjusted model 5: model 4 plus irritability at 3 years | -- | 1.08 (1.01-0.16), p=0.019 | 1.08 (1.02-1.15), p=0.008 |
| Adjusted model 6: model 5 plus irritability at 5 years | -- | -- | 1.07 (1.01-1.14), p=0.029 |

**Table S12. Results of univariable and multivariable models examining the association between a one standard deviation increase in irritability slope from age three to seven years** **(using the three item irritability measure which excludes the item ‘gets over being upset quickly’) and depressive symptoms and self-harm at 14 years in those with complete exposure, outcome and confounder data using population weights (n=7225)**

| **Model** | **Depressive symptoms as a continuous outcome**  **Coef (95% CI), p-value** | **Self-harm at 14 years (binary outcome)**  **Odds ratio (95% CI), p-value** |
| --- | --- | --- |
| Univariable model | 0.15 (0.01-0.31), p=0.037 | 1.06 (0.99-1.13), p=0.075 |
| Adjusted model 1: child sex and child ethnicity | 0.25 (0.11-0.38), p=0.001 | 1.09 (1.03-1.17), p=0.005 |
| Adjusted model 2: model 1 plus maternal age, maternal education, maternal social class, family income quintile, family housing tenure, maternal lifetime depressive symptoms, maternal depressive symptoms at 3 years | 0.25 (0.11-0.39), p=0.001 | 1.10 (1.03-1.17), p=0.005 |
| Adjusted model 3: model 2 plus child school readiness and vocabulary level at age 3 years | 0.24 (0.10-0.39), p=0.001 | 1.09 (1.02-1.17), p=0.008 |
| Adjusted model 4: model 3 plus child emotional, peer, hyperactivity and conduct problems score at age 3 years | 0.28 (0.14-0.41), p<0.001 | 1.10 (1.03-1.18), p=0.003 |
| Adjusted model 5: model 4 plus irritability intercept | 0.29 (0.15-0.43), p<0.001 | 1.11 (1.04-1.18), p=0.002 |
| Adjusted model 6: model 4 plus irritability at 3 years (n=6997) | 0.37 (0.21-0.52), p<0.001 | 1.14 (1.05-1.22), p=0.001 |

## *Using multi-level model predicted childhood irritability at three years (i.e. intercept)*

The predicted estimate of irritability at three years derived from the multi-level model, was not used as an exposure in the main analyses due to the availability of data on irritability at age three years. The actual measurement at age three years was preferable because the value of the predicted intercept estimate is potentially affected by the estimate of the predicted slope, due to nature and assumptions of the multilevel model (which fits a line across the data points) and may therefore be artificially higher or lower than the actual value and therefore provide less accurate/valid results when used in subsequent models.

This can be seen in the models which use the predicted intercept at age three years as the exposure. For depressive symptoms: there was evidence that children with higher intercept scores at three years were associated with higher depressive symptoms at age 14 years in univariable models (n=7225, Coef. 0.41, 95%CI 0.25-0.58, p<0.001). The evidence for this association remained after the inclusion of confounders (Coef. 0.31, 95%CI 0.15-0.47, p<0.001), and following the addition of child development and child emotional and behavioural difficulties as covariates (Coef. 0.23, 95%CI 0.04-0.42, p=0.017). For self-harm: there was evidence that children with higher intercept scores at three years were associated with increased self-harm at age 14 years in univariable models (n=7225, OR 1.16, 95%CI 1.08-1.25, p<0.001). The evidence for this association remained after the inclusion of confounders (OR 1.12, 95%CI 1.03-1.21, p=0.009), and following the addition of child development and child emotional and behavioural difficulties as covariates (OR 1.11, 95%CI 1.01-1.21, p=0.033).

## References

1 Baron A, Malmberg L-E. A vicious or auspicious cycle: The reciprocal relation between harsh parental discipline and children’s self-regulation. *European Journal of Developmental Psychology* 2019; **16**: 302–17.

2 Sorcher LK, Goldstein BL, Finsaas MC, Carlson GA, Klein DN, Dougherty LR. Preschool Irritability Predicts Adolescent Psychopathology and Functional Impairment: A 12-Year Prospective Study. *J Am Acad Child Adolesc Psychiatry* 2021; published online Sept 2. DOI:10.1016/J.JAAC.2021.08.016.

3 Vogel AC, Jackson JJ, Barch DM, Tillman R, Luby JL. Excitability and irritability in preschoolers predicts later psychopathology: The importance of positive and negative emotion dysregulation. 2019. DOI:10.1017/S0954579419000609.

4 Rothman K, Greenland S, Lash T. Validity in epidemiological studies. In: Rothman K, Greenland S, Lash T, eds. Modern Epidemiology. Wolters Kluwer Health/Lippincott Williams & Wilkins, 2008: 128–47.

5 Greenland S, Rothman K, Lash T. Measures of effects and measures of association. In: Rothman K, Greenland S, Lash T, eds. Modern Epidemiology. Wolters Kluwer Health/Lippincott Williams & Wilkins, 2008: 51–70.

6 Kessler R, Andrews G, Colpe L, *et al.* Short screening scales to monitor population prevalences and trends in non-specific psychological distress. *Psychological Medicine* 2002; **32**: 959–76.

7 Elliott CD, Smith P, McCulloch K. British Ability Scales Second Edition (BASII). Technical manual. London, UK, 1997.

8 Goodman MR, Ford T, Simmons H, Gatward R, Meltzer H. Using the Strengths and Difficulties Questionnaire (SDQ) to screen for child psychiatric disorders in a community sample. *The british journal of psychiatry* 2000; **177**: 534–9.

9 Collins LM, Schafer JL, Kam CM. A comparison of inclusive and restrictive strategies in modern missing data  procedures. *Psychological methods* 2001; **6**: 330–51.

10 Johnson DR, Young R. Toward Best Practices in Analyzing Datasets with Missing Data: Comparisons and Recommendations. *Journal of Marriage and Family* 2011; **73**: 926–45.
